# Gene expression analysis and proximity labeling reveal post-transcriptional functions of the yeast RNA polymerase II regulator Def1

**DOI:** 10.1016/j.jbc.2025.111003

**Published:** 2025-12-05

**Authors:** Oluwasegun T. Akinniyi, Shardul Kulkarni, Mikayla M. Hribal, Cheryl A. Keller, Belinda M. Giardine, Joseph C. Reese

**Affiliations:** 1 Department of Biochemistry and Molecular Biology, The Pennsylvania State University, University Park, Pennsylvania, USA; 2 Center for Eukaryotic Gene Regulation, The Pennsylvania State University, University Park, Pennsylvania, USA; 3 Huck Institutes of the Life Sciences Genomics Research Incubator, The Pennsylvania State University, University Park, Pennsylvania, USA

**Keywords:** Def1, RNA polymerase II, transcription, post-transcriptional control, mRNA decay

## Abstract

Def1 is a yeast protein that promotes transcription elongation and regulates the degradation of RNA polymerase II during transcription stress. Although Def1 is localized in the cytoplasm, its functions in this cellular compartment are not yet understood. Despite its well-established roles in transcription, a comprehensive genome-wide analysis of its impact on gene expression has not been conducted. Here, we performed RNA-Seq analysis on cells lacking *DEF1* and surprisingly found that only a few hundred genes exhibited altered expression, both upregulated and downregulated. To evaluate mRNA synthesis and decay rates in these *DEF1*-deficient cells, we used a nascent transcription metabolic labeling technique called RATE-Seq (RNA Approach To Equilibrium sequencing). As expected, we observed reduced synthesis rates across the genome in these cells. In addition, a global decrease in mRNA decay rates was observed, suggesting that Def1 plays a role in the post-transcriptional regulation of mRNAs. The changes in synthesis and decay rates showed a strong correlation, indicating that this compensation helps buffer steady-state mRNA levels. To gain further insight into Def1's functions, we conducted proximity labeling experiments to identify its protein binding partners within the cells. Our findings revealed that Def1 primarily interacts with cytoplasmic regulators involved in post-transcriptional processes, including proteins responsible for deadenylation, decapping, and translation regulation. Using an mRNA decay reporter assay, we demonstrated that recruiting Def1 to mRNA reduces its expression and accelerates its turnover. In summary, we have identified a novel cytoplasmic function for Def1, establishing it as a key regulator of gene expression in both transcription and mRNA decay.

Controlling gene expression involves coordinating multiple processes in both the cytoplasm and the nucleus. One important aspect of this coordination is known as gene expression buffering. This phenomenon occurs when changes in mRNA synthesis within the nucleus are offset by corresponding changes in mRNA decay in the cytoplasm (1, 2, 3, 4). For example, complexes and proteins that were recognized for their impact on transcription in the nucleus can also influence post-transcriptional events in the cytoplasm (2, 3, 5). These proteins are compelling candidates for investigating how various stages of gene expression are coregulated. How the coordination between processes is achieved is unclear, but it involves either the shuttling of these regulators between the nucleus and cytoplasm or indirectly through feedback loops.

DNA-damaging agents disrupt multiple steps in gene expression, affecting transcription, mRNA degradation, and protein synthesis (translation). One cellular response to damaged DNA is the ubiquitylation and subsequent degradation of the large subunit of RNA polymerase II (RNAPII), known as Rpb1 (6, 7, 8, 9). A key protein involved in this process is yeast Degradation Factor 1 (DEF1), which was first identified as a protein that copurifies with the chromatin-bound transcription-coupled repair protein Rad26 (10). Def1 binds to arrested RNAPII and serves as a scaffold, facilitating the recruitment of ubiquitin ligases and other factors necessary for the degradation of RNAPII (6, 9, 11). Def1 undergoes proteasome-mediated proteolytic processing in the cytoplasm, which is dependent on DNA damage and transcription stress (12). This processing separates the N terminus of the protein from its C terminus, which is rich in polyglutamine and helps retain the protein in the cytoplasm. Def1 also plays roles in the nucleus, such as transcription initiation, elongation, telomere maintenance, and various DNA repair pathways (6, 13). Remarkably, despite its established role as a regulator of RNAPII, comprehensive gene expression analyses in *DEF1* mutants have not yet been reported.

While much attention has focused on Def1's nuclear functions, it is an abundant protein found in the cytoplasm. A key question arises: Does Def1 have functions in the cytoplasm, or is its presence in this compartment merely a means of preventing interference with RNAPII transcription in the nucleus under nonstress conditions? Evidence supporting the former hypothesis comes from proximity labeling (BioID) experiments, which have shown that subunits of the Ccr4–Not deadenylase complex interact with Def1 (14). In addition, the presumed human homolog of Def1, UBPA2/2L, has been found to bind to multiple cytoplasmic RNA-binding proteins (15).

In this study, we performed gene expression analysis in conjunction with proximity labeling techniques to investigate the potential cytoplasmic functions of the yeast protein Def1. Our findings reveal that Def1 interacts with several cytoplasmic proteins involved in the post-transcriptional regulation of mRNAs, particularly those regulating mRNA decay. The absence of Def1 results in a significant decrease in mRNA decay rates globally. Importantly, when Def1 is recruited to an mRNA, it accelerates the turnover of that mRNA and suppresses its expression. Therefore, our results suggest that Def1's presence in the cytoplasm is essential not only for preventing interference with transcription in the nucleus but also for regulating mRNA stability and expression.

## Results

### Def1 affects the steady-state RNA levels of a few transcripts

**Figure 1:**
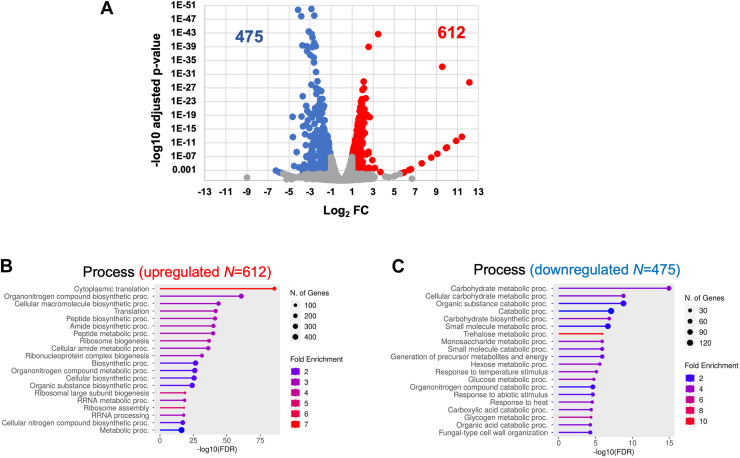
***DEF1* is required for mRNA synthesis and decay.***A,* total RNA-Seq was performed in duplicate for both WT and *def1Δ* strains. DESeq2 was used to determine changes in expression. *Saccharomyces pombe* cells were added as spike-in control before RNA isolation to normalize the data. Upregulated transcripts (>2-fold change, *p*_adj_ < 0.01) appear in *red*, and downregulated transcripts (<2-fold change, *p*_adj_ < 0.01) appear in *blue*. ShinyGO 0.80 was used to determine the enriched biological processes associated with the upregulated (*B*) and downregulated (*C*) genes. *DEF1*, Degradation Factor 1.

Def1 plays a vital role in regulating transcription elongation, particularly by removing stalled RNAPII during transcriptional stress (6, 9, 12). Despite its regulatory function on RNAPII activity, no gene expression analyses of DEF1 mutants have been reported. To address this, we conducted RNA-Seq on WT and *def1Δ* mutant strains. Anticipating that Def1 would have widespread effects on transcription, we included *Saccharomyces pombe* cells as a spike-in control before RNA isolation (see the Experimental procedures section). Two replicates of the experiment were analyzed and displayed a high degree of correlation (*R*^2^ > 0.99) as shown in Figure S1*A*. A total of 6486 RNAs were detected, encompassing all types of RNA. After normalizing the data against the *S. pombe* spike-in reads, we identified differentially expressed genes (DEGs) using a cutoff of >2-fold change and a *p*_adj_ < 0.01. This analysis yielded 612 transcripts that were upregulated and 475 transcripts that were downregulated (Fig. 1*A* and File S1).

Although a variety of RNA types were sequenced, all differentially expressed transcripts were mRNAs (not shown). We performed Gene Ontology (GO) term analysis on the DEGs; the upregulated genes were associated with ribosomal functions, ribosome biogenesis, and translation processes. In contrast, the downregulated transcripts in the mutant were linked to carbohydrate metabolism and various metabolic pathways (Figs. 1, *B* and *C*, and S1, *B* and *C*).

**Figure 2:**
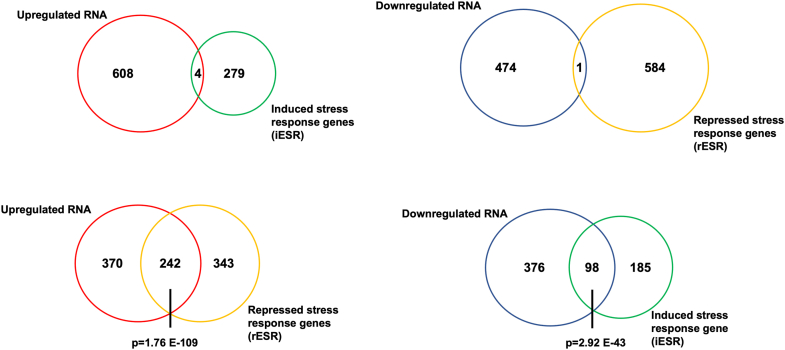
**Comparison of gene expression changes to the environmental stress response (ESR) genes.***Venn diagrams* comparing the overlaps between ESR genes and differentially expressed genes in the *def1Δ* mutant. The collection of induced ESR (iESR) and repressed ESR (rESR) genes (16) was compared to upregulated and downregulated genes in the *def1Δ* mutant strain, as indicated in the figure.

Def1 is involved in DNA damage stress response pathways, and cells lacking this gene (*def1Δ* cells) exhibit slow growth (10, 12). To investigate whether changes in gene expression were due to the cells sensing and responding to stress, we compared the DEGs with the environmental stress response (ESR) genes. ESR genes are regulated by a variety of stressors, including heat shock, DNA damage, nutrient starvation, and oxidative stress, which collectively comprise approximately 900 genes (16). Induced ESR (iESR) genes, or induced, are associated with functions, such as amino acid transport, redox processes, proteasome activity, and detoxification. In contrast, the repressed ESR (rESR) group comprises genes that play roles in transcription and translation, as well as ribosomal proteins and those involved in ribosome biogenesis. Our analysis revealed minimal overlap between the upregulated and downregulated DEGs with the iESR and rESR, respectively (Fig. 2). This finding suggests that the changes in gene expression in *def1Δ* cells are not a result of cellular stress. On the other hand, the opposite was observed. Upregulated DEGs in the *def1Δ* mutant overlapped considerably with the rESR, and the downregulated DEGs overlapped with the iESR (Fig. 2). As mentioned above, the upregulated DEGs are enriched with genes that regulate translation and ribosome biogenesis, and these classes of genes are enriched in the rESR gene set.

### *DEF1* maintains mRNA synthesis and decay rates

Def1 is recognized for its role in both the initiation and elongation phases of transcription. Therefore, we were surprised to observe so few changes in gene expression, particularly the number with reduced expression levels. RNA-Seq measures the steady-state levels of RNA, which is a function of both synthesis and decay. Previous studies have shown that cells can adjust one pathway in response to changes in another to maintain steady-state mRNA levels, a phenomenon known as transcript buffering (1, 4, 17). We therefore investigated the rates of transcript synthesis and decay in the *def1Δ* mutant. We utilized the RNA Approach To Equilibrium sequencing (RATE-Seq) method, which is a pulse-only metabolic labeling technique. In this method, cells are treated with 4-thiouracil (4tU) for varying labeling durations as the incorporation of the nucleoside analog approaches equilibrium (18). This method accounts for a strain's doubling time to compensate for slow-growing strains. 4tU-labeled *S. pombe* RNA was used as a spike-in control to normalize between samples and to account for widespread changes in gene expression (see the Experimental procedures section). We conducted RATE-Seq on both WT and *def1Δ* cells to estimate synthesis and decay rates for 3938 and 5631 transcripts, respectively. The data from the replicates showed a very high level of correlation (Fig. S2).

**Figure 3:**
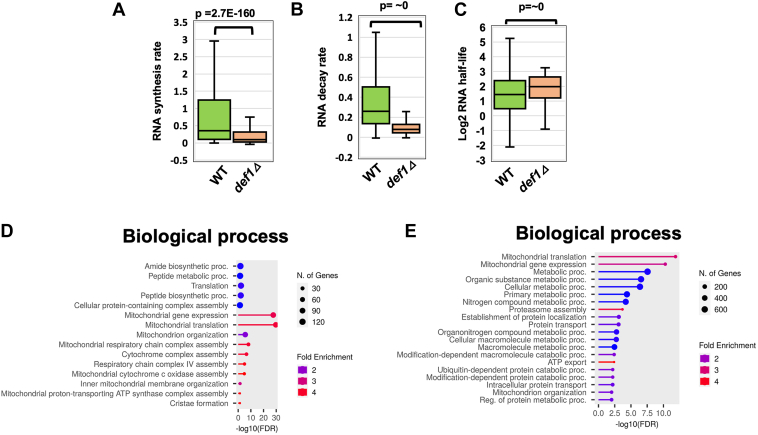
**Def1 regulates rates of mRNA synthesis and degradation.***A,* RNA synthesis rates are reduced in the *def1Δ* mutant. RATE-Seq was used to determine the global rates of RNA synthesis. Rates obtained for the 3596 transcripts that overlap in the WT and *def1Δ* samples are displayed in the figure. The *p* value was determined using the Wilcoxon rank sum test. *Y*-axis is molecules/min. *B,* Def1 globally controls mRNA decay rates. As in “*A,*” except decay rates were plotted. *C,* as in “*A*,” except decay rates were used to estimate mRNA half-lives. *D,* Gene Ontology (GO) terms for the biological process of the top 20th percentile of the genes with the highest magnitude of reduced nascent RNA synthesis (*def1Δ*/WT). GO terms were determined using ShinyGO 0.80. *E,* GO terms for the biological processes of the top 20th percentile of mRNAs with the largest change in mRNA decay. *Def1*, Degradation Factor 1; RATE-Seq, RNA Approach To Equilibrium sequencing.

We analyzed the median synthesis and decay rates of 3596 transcripts that were common to both the WT and mutant datasets. In the mutant cells, the global mRNA synthesis rate was significantly reduced. Specifically, the median synthesis rate in the mutant was approximately 3.7-fold lower than that in WT cells; however, for some transcripts, the change in synthesis was much more pronounced (Fig. 3*A* and File S2). Given that the overall synthesis rates were reduced, yet relatively fewer transcripts exhibited lower expression, this suggested that RNA degradation rates were reduced in the mutant. We calculated the decay rates and RNA half-lives and found that, under the experimental conditions, the median decay rate in WT cells was 0.259 molecules/min, which corresponds to a median half-life of 2.7 min (Fig. 3, *B* and *C* and File S2). Our estimated half-life is shorter than some previously reported measurements; however, the half-life values found in other studies have varied between 2 and 18 min and are highly dependent on the methodology and labeling conditions used (Fig. S3). Most importantly, we found that the decay rate in the mutant was 3.2-fold lower, indicating a global stabilization of mRNAs in these cells (Fig. 3, *B* and *C*).

If Def1 regulates the RNAs of genes involved in specific cellular processes or pathways, we anticipate that its effects on mRNA metabolism will be most significant for those transcripts. To investigate whether Def1 targets mRNAs within a particular pathway, we performed GO term analysis on the transcripts in the top 20th percentile for synthesis (Fig. 3*D*) and half-life fold change (Fig. 3*E*). The biological processes most affected by the deletion of *DEF1* included mitochondrial translation, protein metabolism, and translation.

**Figure 4:**
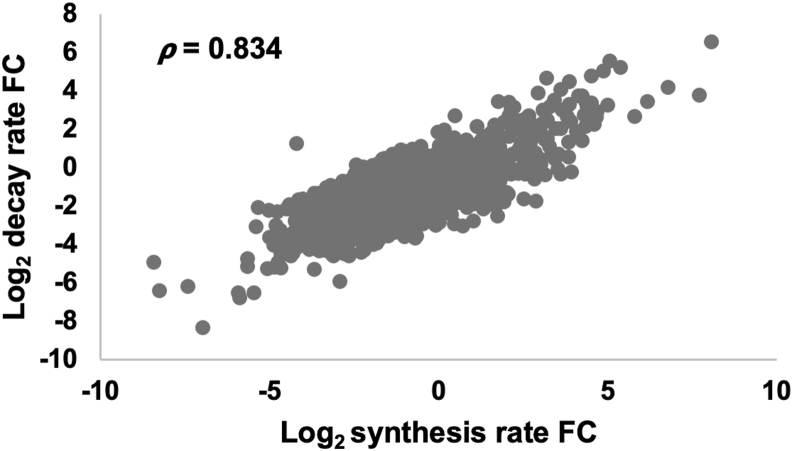
**The *def1Δ* mutant displays gene expression buffering.** A scatterplot was constructed by plotting the fold change (FC) in RNA decay and synthesis rates plotted on the *y*- and *x*-axis, respectively. The Spearman's correlation coefficient is shown in the scatterplot.

We then plotted the changes in decay rates of individual mRNAs against their changes in synthesis rates (Fig. 4). We observed a strong positive correlation (*R* = 0.834), which indicates that transcript buffering is occurring in the mutant. This suggests that Def1 is not necessary for transcript buffering, and the corresponding changes in synthesis and decay rates explain why no global (widespread) change in steady-state mRNA levels was observed in the mutant.

### Def1 interacts with cytoplasmic mRNA regulatory proteins

Proximity labeling, using an engineered biotin ligase (BioID), is a technique employed to identify protein–protein interactions within cells, allowing for the detection of transient and weak interactions (19, 20). Recently, we conducted BioID experiments on subunits of the Ccr4–Not complex and discovered that several bait proteins strongly labeled Def1 (14). In addition, the human homolog of Def1, UBAP2/2L, was found to interact with hCcr4–Not subunits (15). Therefore, we performed a reciprocal experiment by fusing the TurboID (TID) version of the biotin ligase enzyme to the C terminus of Def1 (21). Deleting *DEF1* causes slow growth and increased sensitivity to DNA-damaging agents (10, 12). We verified that the fusion of TID to the C terminus of Def1 did not affect cell growth, even in the presence of hydroxyurea, suggesting that the addition of TID to Def1 is well tolerated (Fig. S4, *A*).

**Figure 5:**
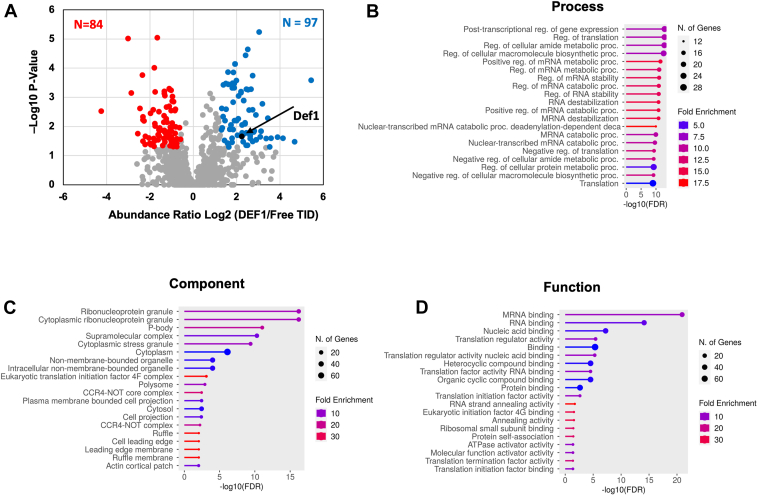
**BioID analysis reveals that Def1 interacts with cytoplasmic post-transcriptional regulatory factors.***A,* BioID labeling was performed in cells expressing Def1–TurboID (TID) and cells expressing free TID enzyme. Biological replicates were analyzed. Biotinylated proteins pulled down by streptavidin beads were identified by mass spectrometry. Data are presented as a Volcano plot of the fold change in protein abundance ratio of Def1–TID *versus* free TID (Log2) *versus* -log10 *p* value. The enriched proteins (>1.5-fold, *p* < 0.05) appear in *blue*, and the under-represented proteins (>-1.5-fold, *p* < 0.05) appear in *red*. Proteins enriched in the Def1–TID sample were analyzed using ShinyGO 0.80, and biological process (*B*), component (*C*), and function (*D*) are shown. *Def1*, Degradation Factor 1.

We expressed “free” TID at levels close to those of Def1–TID as a control (Fig. S4, *B* and *C*). The interacting proteins were identified by calculating the fold enrichment of Def1–TID-labeled proteins *versus* free TID. Labeled proteins were subjected to mass spectrometry, and we implemented a cutoff of 1.5-fold enrichment and *p* < 0.05 to identify interacting or proximal partners of Def1 (Fig. 5*A* and Supplementary File 3). A total of 97 proteins met these criteria, with 42 involved in various aspects of RNA regulation. Notably, Def1–TID also labeled retrotransposon proteins (Ty), which were not included in the 97 proteins (File. S3). The significance of this is not known. We submitted the enriched proteins to GO term analysis, which returned terms associated with the regulation of translation, post-transcriptional control, and mRNA decay. The analysis for cellular component yielded terms, such as ribonucleoprotein (RNP) granule, P-body, translation initiation factor 4F, polysome, and the Ccr4–Not core complex (Fig. 5, *B*–*D*). Overall, Def1–TID predominantly labeled cytoplasmic RNA regulators.

Eighty-four proteins were found to be under-represented in the Def1–TID sample compared with the free TID control sample. Previous BioID studies have shown that proteins can be under-represented in the test sample relative to the free control (14). This under-representation may be due to the fusion of the test protein to TID, which might prevent the TID enzyme from labeling nontargets nonspecifically within the cell. When we subjected the under-represented proteins to GO term analysis, we found broad terms with weak false discovery rates and enrichment scores (not shown), suggesting a lack of biological significance.

It was surprising that Def1 did not label many RNAPII transcription factors or other nuclear proteins involved in mRNA synthesis. The predominant localization of Def1 in the cytoplasm, particularly in the absence of transcription stress, likely explains why few nuclear proteins were labeled. In addition, the biotin ligase was fused to the C terminus of Def1, and the C terminus retains Def1 in the cytoplasm (12). These data suggest that Def1 interacts with cytoplasmic RNA–regulating proteins, indicating that it may have post-transcriptional functions, such as mRNA storage, translation, or decay.

### Tethering Def1 directly to an mRNA reduces its expression and accelerates its decay

**Figure 6:**
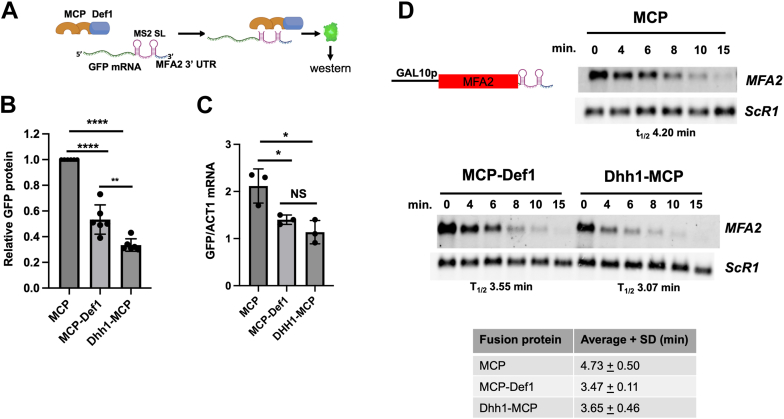
**MS2–MCP tethering reporter assays suggest that Def1 functions in post-transcriptional control of mRNAs.***A,* schematic of the MCP–MS2 tethering assay. MCP–Def1 has MCP fused to the N terminus; Dhh1–MCP was used as a positive control. Expression of the free MCP and the MCP–Def1 fusion proteins was driven by the *DEF1* promoter. Dhh1–MCP was driven by the *DHH1* promoter. *B,* the relative abundance of GFP protein in the fusion protein–expressing cells, compared with that expressing free MCP protein, was set at 1.0. Taf14 was used as a loading control, and GFP protein signals were normalized to the loading control. The averages of three biological replicates from two experiments are shown (N = 6). The error bars represent the standard deviations. *p* Values were calculated using an unpaired *t* test. ∗∗∗∗*p* < 0.0001; ∗∗*p* < 0.01. *C,* graph of steady-state GFP mRNA. RT–quantitative PCR measured the abundance of both GFP and *ACT1* (control) mRNA. GFP mRNA amounts were normalized to *ACT1*. Biological triplicates were analyzed (N = 3). *p* Values were calculated using an unpaired *t* test. ∗*p* < 0.05 (*D*, *upper*). Cells transformed with a GAL10p-MFA2-MS2 reporter gene (22) and each of the MCP derivatives described in the panel were grown in galactose-containing dropout medium, transferred to dextrose-containing medium, and samples were collected at various time points following transcription shutdown. The levels of *MFA2* RNA were detected by Northern blotting. *ScR1* was the loading control. The calculated half-lives, after correcting for loading, are listed below the panel. *D, lower, MFA2* reporter mRNA half-lives from multiple determinations (N = 4). The signals from *MFA2* were normalized to those of the loading control *ScR1*. The averages and standard deviations of reporter mRNA half-lives are reported. *p* Values were calculated using a two-tailed unpaired *t* test. The p Values comparing the half-life between cells expressing MCP and MCP–Def1 and Dhh1–MCP were *p* < 0.01 and *p* < 0.054, respectively. *Def1*, Degradation Factor 1; MCP, MS2 coat protein.

The extended half-lives of mRNA in the *def1Δ* mutant, along with the labeling of various mRNA deadenylation and decapping factors by Def1–TID, suggest that Def1 regulates mRNAs in the cytoplasm. However, the decreased mRNA turnover observed could be a compensatory response to a lower synthesis rate caused by transcript buffering (1, 4). To provide evidence that Def1 regulates mRNA turnover, we employed an MS2 coat protein (MCP)/MS2-tethered reporter assay, which has been previously used to demonstrate the direct regulation of mRNA by decay factors (22). The reporter construct produces an mRNA that includes an ORF for GFP along with MS2 stem loops in the 3′ UTR (Fig. 6*A*). We fused MCP to the N terminus of Def1 (labeled as MCP–Def1). The MCP–Def1 fusion protein complemented a *def1Δ* strain, demonstrating its functionality (Fig. S5, *A*). The expression levels of the MCP–Def1 fusion protein were similar to those of the free MCP protein (Fig. S5, *B* and *C*). As a positive control, we tested a Dhh1–MCP fusion protein, which has been previously shown to repress reporter mRNA (22, 23). We measured the levels of GFP protein produced by the reporter. Tethering Def1 to the mRNA resulted in a significant decrease in GFP protein levels compared with the free MCP control (Fig. 6*B*). Specifically, GFP protein levels were reduced to approximately 50% in cells expressing the Def1 fusion protein, although this reduction was not as pronounced as the repression observed with the Dhh1–MCP control. Next, we assessed mRNA levels using quantitative PCR (qPCR). The amount of GFP mRNA was normalized to that of *ACT1* and plotted (Fig. 6*C*). The results showed that the mRNA levels of the reporter were lower in cells expressing MCP–Def1 compared with those expressing MCP alone. Furthermore, Dhh1–MCP was slightly more effective at repressing the reporter mRNA expression.

We investigated whether tethering Def1 to an mRNA could reduce the half-life of an RNA reporter. To achieve this, we used a reporter construct that included the MFA2 ORF followed by a 3′ UTR containing MS2-binding sites, all under the control of the *GAL10* promoter. We shut down transcription by shifting the cells from a medium containing galactose to one with dextrose. Figure 6*D* presents a representative experiment and the average results from multiple experiments (N = 4). In cells expressing only the MCP protein, the reporter mRNA had an average half-life of approximately 4.2 min. Conversely, cells expressing the MCP–Def1 fusion protein showed a significantly greater enhancement in decay, reducing the average half-life to about 3.6 min (*p* < 0.01). The reduction in half-life observed with the MCP–Def1 fusion was comparable to the Dhh1 control, which had an average half-life of around 3.7 min (*p* < 0.054). These reporter assays provide evidence that Def1 plays a role in regulating mRNAs post-transcriptionally.

Def1 functions similarly to other factors involved in both the decay and synthesis of mRNAs. Previous studies have demonstrated that tethering mRNA decay factors or general transcription factors to promoters can activate transcription (2, 24). Def1 copurified with the general transcription factor TFIIH was crosslinked to promoters of genes and affected start site selection in an *in vitro* transcription assay, suggesting it may regulate initiation (13). Def1 has been shown to copurify with the general transcription factor TFIIH and influence start site selection in *in vitro* transcription assays (13). Given this information, we tested whether recruiting Def1 to a promoter would enhance transcription. A reporter gene containing β-galactosidase under the control of eight LexA operators was used. We constructed fusion proteins combining the DNA-binding domain of LexA with full-length Def1. The expression of both LexA alone and the LexA–Def1 fusion was driven by the *DEF1* promoter, as described in the *Experimental procedures* section. For comparison, we also analyzed the LexA–TBP (TATA-binding protein) fusion as a positive control. While the LexA–TBP fusion strongly activated the reporter gene, the LexA–Def1 protein did not (Fig. S6). However, it is important to note that this tethering assay assesses the effects on preinitiation complex formation, and a negative result does not rule out Def1's potential impact on transcription, particularly during the elongation phase.

## Discussion

An increasingly large number of gene regulatory proteins are being discovered to have new or previously overlooked functions. For instance, Ccr4–Not, originally believed to regulate TBP and transcription initiation primarily, is now acknowledged for its significant role in the post-transcriptional control of mRNAs. In addition, Def1 has been identified as a key player in mitigating DNA damage. Extensive research has established Def1 as a factor that facilitates the elongation process and degrades Rpb1, thereby clearing stalled RNAPII from genes. Other nuclear functions have also been attributed to Def1 (for review, see Ref. (6)). Excluding Def1 from the nuclear compartment is crucial to prevent interference with transcription (as noted in Ref. (12)). Although Def1 is present in the cytoplasm, its cytoplasmic functions have not been identified. In this study, we report a new role for Def1 in the cytoplasm related to post-transcriptional gene control.

Def1 has long been recognized as an RNAPII elongation factor, copurifying with TFIIH and influencing start site selection in reconstituted transcription reactions (6, 12, 13). Our study describes the first transcriptome analysis of this transcription factor. Consistent with its expected role in transcription, the deletion of *DEF1* globally reduced mRNA synthesis (Fig. 3*A*). Its function in maintaining mRNA decay rates was unexpected, suggesting a role in the cytoplasm. While the reduced synthesis rates in the mutant may contribute to the slowed mRNA decay because of transcript buffering, we present additional evidence that Def1 plays a direct role in mRNA decay. First, Def1 binds many cytoplasmic post-transcriptional regulators, including the major deadenylase complex, Ccr4–Not, as demonstrated by BioID labeling. Both Def1 and Ccr4–Not are involved in regulating transcription elongation and the degradation of Rpb1 during DNA damage stress, which could have explained their interaction (6, 12, 25). However, evidence for a function in decay also comes from the MS2–MCP tethering reporter assays. The recruitment of Def1 to an mRNA accelerated its decay and repressed its expression. Moreover, proximity labeling experiments using Def1, Not1, and Not4 as baits revealed reciprocal interactions in cells (Fig. 5 and (14)). This suggests that Def1's ability to control mRNA decay may be mediated by recruiting decay factors, such as Ccr4–Not, to mRNAs. We attempted to examine this by conducting the MS2–MCP reporter assay in a *ccr4Δ* mutant. However, we found that the expression of Def1–MCP protein in the *ccr4Δ* mutant was severely compromised, reduced to only 20% of the levels observed in WT cells (not shown), which made the assay results difficult to interpret. In addition, Def1 also labeled the decapping enzyme Dcp2, indicating that Def1 could influence this step in mRNA degradation as well. Further studies are needed to clarify how Def1 regulates these processes. The recruitment of decay factors may involve the polyglutamine-rich C terminus of the protein, as this is the location where the TID enzyme was fused.

A cryptic nucleic acid–binding domain has been identified in Def1, specifically between positions 1 and 207 (26). This domain may bind to transcripts nonspecifically; however, it is more probable that Def1 is recruited to mRNAs *via* the Ccr4–Not complex or through sequence-specific RNA-binding proteins. The Def1–TID protein biotinylated multiple sequence–specific RNA-binding proteins, including all members of the Pumilio family of RNA-binding proteins (Puf1/Jsn1, Puf2, Puf3, and Puf4) (Supplemental File 3). These proteins bind to specific sequences in the 3′ UTRs of RNAs, marking them for degradation and translational repression (27). Interestingly, Puf3 is involved in targeting and promoting the degradation of mRNAs of nuclear-encoded mitochondrial proteins (28, 29), and the mRNAs that are most sensitive to the loss of Def1 encode for mitochondrial proteins (Fig. 3, *D* and *E*).

Def1 interacts with RNP granule markers, including Ccr4–Not subunits, Edc3, Dhh1, and Lsm7. RNP granules, such as stress granules and processing bodies, are cytosolic compartments that facilitate translational repression, where mRNAs are stored alongside initiation and decay factors during times of stress (30). However, neither UV stress (12) nor nutrient stress (data not shown) induced the formation of Def1-containing foci, such as processing bodies. Recently, UBAP2/2L was identified as the putative human homolog of Def1, as it has been shown to direct the ubiquitylation of the large subunit of RNAPII, Rpb1 (31). UBAP2/2L is located in stress granules and is necessary for their formation (32, 33). While UBAP2/2L possesses a ubiquitin-binding domain similar to that of Def1, it lacks the polyQ-rich domain found in Def1. Therefore, it was unclear whether Def1 has cytoplasmic functions or if UBAP2/2L acquired these functions. Although Def1 does not form detectable cytoplasmic foci, it may regulate the partitioning of mRNAs between the repressed pool and the actively translated pool. Not4 and Not5, members of the Ccr4–Not complex, play a crucial role in this partitioning, determining the distribution of mRNAs between the soluble and insoluble fractions (34).

Our research addressed the perplexing cytoplasmic localization and the remarkably high abundance of Def1. According to the *Saccharomyces cerevisiae* database, the median abundance of Def1 is estimated to be around 21,000, but some estimates predict the copy number as high as 80,000 per cell. As mentioned earlier, DNA damage leads to the proteasomal processing of a portion of Def1, whereas a larger fraction of the unprocessed protein remains in the cytoplasm. Def1 has functions in the cytoplasm, and its presence in this part of the cell serves a purpose beyond merely preventing interference with transcription in the nucleus, as previously suggested (12). Def1 contributes to both transcription and crucial cytoplasmic functions, thereby adding it to the list of factors involved in transcription within the nucleus, as well as mRNA decay in the cytoplasm. Therefore, Def1 may play a vital role in nuclear–cytoplasmic communication by coordinating transcription and decay.

## Experimental procedures

### Yeast strains and plasmids

Strains and plasmids used in this work are contained in Tables S1 and S2, respectively. Cells were grown in rich media, 2% bactopeptone, 1% yeast extract, 20 μg/ml adenine sulfate, and 2% dextrose or synthetic dropout media made with yeast nitrogen base plus ammonium sulfate and either 2% galactose or dextrose. Gene deletions were produced by homologous recombination using PCR-generated cassettes (35). The genotypes of the mutants were screened by PCR of genomic DNA. Plasmids were sequenced. Details on the construction are available upon request.

### Growth spot assay

Yeast strains were grown to saturation at 30 °C in 5 ml of the appropriate medium. Serial dilutions were prepared in sterile distilled water, and diluted samples were spotted on plates containing the appropriate media and supplements. Plates were incubated at 30 °C for 24 h or more, as indicated in the respective figures.

### Western blotting

Samples were separated on either SDS-PAGE or Tris–acetate gradient gels, and proteins were transferred to nitrocellulose membranes by semidry transfer. Membranes were stained with Ponceau S, imaged, and then destained. Membranes were blocked in 5% milk in Tris-buffered saline with Tween-20 (50 mM Tris–HCl, pH 7.5, 0.15 M NaCl, 0.1 mM EDTA, and 0.1% Tween-20) for at least 1 h and then probed with the primary antibody in 2% or 4% milk for at least 2 h. Antibodies to Taf14 and Dhh1 have been described in other publications and are custom antibodies raised in rabbits, validated by analyzing an extract from cells with the gene deleted (14, 36). Antibodies to Def1 were raised in rabbits to a 6HIS-Def1 (1–500) fusion. The Def1 was validated by probing an extract from a *def1Δ* strain. Commercial primary antibodies used are Anti-FLAG M2 (MilliporeSigma), GFP (Y1030; UBP-Bio), and anti-HA (HA.11; BioLegend). Epitope tag antibodies were validated by including a sample from untagged cells and observing mobility changes in proteins of different sizes. Signals were detected by fluorescence or chemiluminescence (enhanced chemiluminescence) and imaged on X-ray film or a ChemiDoc MP Imaging System (Bio-Rad). Statistical analysis and graph construction were performed in GraphPad Prism (GraphPad Software, Inc).

### BioID

TID was fused to the C terminus of Def1 and truncation derivatives, using PCR-derived cassettes from pJR106 as described (14). Free TID was expressed from the *CHA1* promoter, and this strain was used as the control. Protein extraction and isolation on streptavidin magnetic beads (Pierce, ThermoFisher, catalog no.: 88817) was conducted as described (14). Biological duplicates were analyzed. Proteins were digested on the beads with 1 μg of trypsin (Promega Trypsin Gold) after alkylation with iodoacetamide overnight at 37 °C. Purified peptides were labeled with TMTpro 16plex reagents (ThermoFisher, catalog no.: A44520) per the manufacturer's recommended protocol. Peptides were analyzed on a Nano-Flow Liquid Chromatography (Thermo Easy-nLC 1200). The peptides were loaded on an Acclaim PepMap100 trapping column (75 mm × 2 cm, C18, 5 mm, 100 Å; Thermo) and separated on an Acclaim PepMap RSLC column (50 mm × 15 cm, C18, 2 mm, 100 Å; Thermo) at a flow rate of 300 nl/min in the following gradient of mobile phase B (80% acetonitrile in 0.1% aqueous formic acid): 5% to 50% B in 210 min, 50% to 90% B in 30 min. A Thermo Orbitrap Eclipse mass spectrometer was operated in a data-dependent mode using a method based on the tandem mass tag MS2 template. The resulting mass spectra were processed using Thermo Proteome Discoverer 3.1 software.

### Total RNA-Seq for steady-state mRNA levels

Cells were grown in 2% bactopeptone, 1% yeast extract, 20 μg/ml adenine sulfate, and 2% dextrose and harvested at log phase (absorbance at 600 nm = 0.8–1). Cells were washed with nuclease-free water and resuspended in AE buffer (50 mM sodium acetate [pH 5.3], 10 mM EDTA, 1% SDS). Cells were spiked with *S. pombe* cells at a ratio of 14:1. RNA was isolated using AE–phenol chloroform extraction, followed by overnight ethanol precipitation with sodium acetate. RNA samples were washed with 80% ethanol and resuspended in nuclease-free water. RNA was quantified on a Nanodrop, and the quality was verified by analysis on a Tapestation. RNA samples with RNA integrity number scores >9.0 were used. Biological replicates were analyzed.

### RNA metabolic labeling studies

The procedure is a derivative of the one used in a previous publication (37). *S. cerevisiae* cells growing in yeast extract, peptone, and dextrose were treated at log phase (absorbance at 600 nm = 0.8–1) for 6, 9, 12, 24, and 90 min with 0.65 mg/ml 4tU (MilliporeSigma). Thirty absorbance at 600 nm cultures were transferred to two volumes of ice-cold methanol on dry ice to halt 4tU incorporation. Cells were harvested by centrifugation at 3000 rpm for 3 min and resuspended in AE buffer (50 mM sodium acetate [pH 5.3], 10 mM EDTA, and 1% SDS). The *S*. *cerevisiae* cells were spiked with prelabeled *S. pombe* cells at a ratio of 1:14. *S. pombe* spike-in was generated by growing cells in yeast extract, peptone, dextrose to log phase (absorbance at 600 nm = 0.8) and treated with 5 mM 4tU for 12 min. RNA was isolated using AE–phenol, then extracted with chloroform, and finally precipitated overnight in ethanol. RNA samples were resuspended in nuclease-free water. RNA was subjected to biotinylation (120 μg RNA, 20 mM Hepes, pH 7.4, 12 μg MTSEA Biotin-XX [Biotium Co] in a final volume of 600 μl for 30 min at room temperature). Free MTS–biotin reagent was removed by phenol–chloroform–isoamyl alcohol extraction and precipitation with NaCl and isopropanol. Dynabead MyOne streptavidin C1 beads (ThermoFisher) were washed twice in high salt wash buffer (HSWB) (100 mM Tris–HCl [pH 7.4], 10 mM EDTA, 1 M NaCl) and blocked with blocking buffer (100 mM Tris–HCl [pH 7.4], 10 mM EDTA, 1 M NaCl, and 40 ng/μl glycogen), then resuspended in HSWB. Eighty micrograms of biotinylated RNA were incubated at 65 °C for 7 min, and a streptavidin pull down was performed using 100 μl blocked beads and then washed thrice with HSWB and eluted twice in 25 μl elution buffer (100 mM DTT, 10 mM EDTA, 1 M NaCl, and 0.05% Tween-20). The RNA was concentrated and quantified on a Qubit instrument (Life Technologies).

### Library preparation and NextGen sequencing

Total RNA was depleted of rRNA using the Qiagen FastSelect (catalog no.: 334215) kit. Libraries for steady-state RNA and RATE-Seq samples were prepared using the Illumina Total Stranded Library Prep kit and the Illumina UDI indexes (catalog no.: 20040529). The libraries were analyzed on TapeStation-4150 at the Genomics Core Facility, Penn State University, and were quantified using NEBNext Library Quant Kit for Illumina. Libraries were sequenced on the Illumina NextSeq 2000. Illumina P2 100-cycle reagent cartridge (#EC1591461-EC11) was used for paired-end sequencing. Reads were mapped to the sacCer3 genome using the ENCODE 3 pipeline. Spike-in reads were mapped to the *S. pombe* genome (https://www.encodeproject.org/pipelines/ENCPL002LPE/). Star was used to map the reads, and RSEM-1.2.28 was used to get estimated counts and transcripts per million (gene: Saccharomyces_cerevisiae.R64-1-1.106.ucsc.gtf). DESeq2 was used to determine the differential expression of genes (38). The truncated expected counts from the RSEM output were used as input for DESeq2. Genes with <15 reads across samples were filtered out. The *p*_adj_ value of 0.01 was used as a threshold.

### RATE-Seq data modeling

RNA reads obtained from DeSeq2 were used for nonlinear regression modeling to obtain decay rates in R using the *nls.table* function. The model for RNA decay is (18):

$$Y_{t}=Y_{eq}\left(1-e^{-\left(\propto_{\text{RNA}}-\propto_{\text{growth}}\right)\left(t\right)}\right)$$

where

Y_t_ = Amount of labeled transcript at time *t*.

Y_eq_ = Abundance of labeled transcript at a steady state

t = Labeling time

RNA decay rate constant, $\propto =\left(\propto_{\text{RNA}}-\propto_{\text{growth}}\right)$

αRNA = RNA decay rate

αgrowth = Growth rate = ln2/doubling time = 0.0077 (WT) or 0.0046 (def1Δ)

Synthesis rate was estimated in Excel from the decay rate constant using the formula:

Synthesisrate=∝[mRNA]

where mRNA abundance is estimated from steady-state read counts, assuming a total of 60,000 mRNA molecules per cell (29).

mRNA half-life was estimated in Excel from the decay rate constant using the formula:

$$\text{Half}-\text{life}=\frac{\ln\;2}{\propto }$$

### mRNA decay and transcription reporter assays

The MS2–MCP mRNA reporter assay was conducted as described in a previous publication (22). Plasmids expressing MCP fusions with Def1 were constructed from gene synthesis and PCR-generated fragments using in-fusion assembly (Takara Bio) (Table S2). Fusion proteins were driven by the *DEF1* promoter and contained its 3′UTR. MCP fusion protein expression plasmids were cotransformed with the reporter vector pJC428 or pJC2 into W303 cells (Table S1). Cells were grown in triplicate in -uracil/-leucine dropout media to an absorbance of 1.0 at 600 nm. The expression levels of the MCP fusion proteins were verified using anti-FLAG antibodies and the levels of the reporter GFP protein using anti-GFP antibodies. To calculate the levels of GFP protein, a serial dilution standard curve of an extract was included on the blots. Loading was normalized to Taf14 levels, which served as a loading control. Signals from images were quantified using ImageJ (National Institutes of Health). An unpaired *t* test was used to calculate the *p* values. Steady-state RNA levels of GFP and *ACT1* were quantified by qPCR using primers described (22). Statistical analysis and graph construction were performed in GraphPad Prism.

mRNA half-life measurements were conducted using a GAL10p-MFA2-MS2 reporter plasmid (22). To measure the half-life of the reporter mRNA, cells were grown to midlog phase in dropout media containing 2% galactose and then collected by centrifugation. After resuspension in dropout media lacking a carbon source, an aliquot was removed and quenched with a half volume of methanol chilled to −70 °C. Dextrose was then added to 4%, and aliquots were removed at different time points and quenched in methanol chilled to −60 °C. RNA was subjected to Northern blotting using random-primed biotinylated DNA probes (BioPrime Kit; ThermoFisher) to detect *MFA2*. After washing, RNA was detected using streptavidin–IR800 (catalog no.: 926-32230; Li-Cor) and scanned on a Chemidoc-MP (Bio-Rad). Blots were stripped of the *MFA2* probe and then probed with *ScR1* as a loading control.

The beta-galactosidase transcription reporter assay was conducted by cotransforming W303 cells with pSH18-34 and plasmids expressing LexA fusion proteins with Def1 (Table S2). Fusion proteins were driven by the DEF1 promoter and contained its 3′UTR. Cells were grown in triplicate in -uracil/-leucine dropout media containing 2% dextrose to an absorbance of 1.0 at 600 nm. Protein extraction and beta-galactosidase assays were performed as described in a previous publication (39).

### RT–qPCR

RNA samples were quantified on a Nanodrop and run on 1% agarose gel to confirm the quality. One microgram of RNA was treated with 1 U DNaseI for 30 min at 37 °C, followed by quenching with 5 mM EDTA at 65 °C for 10 min. RevertAid RT kit (ThermoFisher; catalog no.: FERK1622) was used for complementary DNA preparation according to the manufacturer's protocol using a mix of random and oligo dT primers. Serial dilutions of complementary DNA were used to set up qPCRs. PCRs were performed with PerfeCTa SYBR Green SuperMix and using an Agilent real-time machine. The data were analyzed using the Agilent AriaMx software and Excel.

## Data availability

Genomics data have been deposited to Gene Expression Omnibus under accession number GSE290861. Proteomics data were uploaded to MassIVE under MSV000097607.

## Supporting information

This article contains supporting information (12, 14, 18, 22, 24, 25, 40, 41, 42, 43, 44, 45, 46, 47).

## Conflict of interest

The authors declare that they have no conflicts of interest with the contents of this article.
