# Supplementary material for: Gene expression analysis and proximity labeling reveal post-transcriptional functions of the yeast RNA polymerase II regulator Def1
Source: J Biol Chem. 2025 Dec 5;302(2):111003. doi: 10.1016/j.jbc.2025.111003 (PMC12804109; doi:10.1016/j.jbc.2025.111003)
Supplement: Supplemental Table 1 [file mmc1.docx]

**Supplemental Table 1. Yeast strains**

| BY4741 | MAT a his3Δ1; leu2Δ0; met15Δ0; ura3Δ0 |  |
| --- | --- | --- |
| BY4742 | MAT alpha his3∆1; leu2∆0; lys2∆0; ura3∆0 |  |
| W303 | MAT alpha, RAD5+, ade2-11; his3-11,15; leu2-3,112; ura3-1; trp1-1; can1-100 | Zhao,X. 2002 |
| JR1951 | BY4742, CHA1p-TID-3HA::KanMx | Pfannenstein,2024 |
| JR2200 | BY4742, DEF1 (1-738)-TID-3HA:: KanMX | This study |
| JR2204 | W303, def1∆::KanMx | This study |

**References:**

Zhao X, Rothstein R. The Dun1 checkpoint kinase phosphorylates and regulates the ribonucleotide reductase inhibitor Sml1. Proc Natl Acad Sci U S A. 2002 Mar 19;99(6):3746-51.

Pfannenstein,J., Tyryshkin,M., Gulden,M.E., Doud,E.H., Mosley,A.L. and Reese,J.C. (2024) Characterization of BioID tagging systems in budding yeast and exploring the interactome of the Ccr4-Not complex. *G3 (Bethesda)*, **14**.
