# Supplementary material for: Gene expression analysis and proximity labeling reveal post-transcriptional functions of the yeast RNA polymerase II regulator Def1
Source: J Biol Chem. 2025 Dec 5;302(2):111003. doi: 10.1016/j.jbc.2025.111003 (PMC12804109; doi:10.1016/j.jbc.2025.111003)
Supplement: Supplemental Table 2 [file mmc2.docx]

| # | features | description | reference |
| --- | --- | --- | --- |
| pJC428 | M/GFP-MS2-URA3 | GFP reporter | Sweet, 2012 |
| pJC2 | pGAL10-MFA2-MS2-URA3 | MFA2-MS2 reporter | Sweet, 2012 |
| pJC236 | DHH1-MS2-LEU2 | DHH1-MCP fusion | Sweet, 2012 |
| pJR106 | pFA6a-TID-HA3-KanMX6 | Turbo ID (TID) tagging | Pfannenstein, 2024 |
| pJR160 | pRS415- DEF1pr-MCP-FLAG3-DEF1 3’UTR | Free MCP expressed from the DEF1 promoter | This study |
| pJR162 | pRS415-MCP-DEF1-FLAG3-DEF1 3’UTR | DEF1-MCP (N-terminus) | This study |
| pJR164 | pRS415- DEF1pr-LexA-FLAG3-DEF1 UTR | Free LexA expressed from the DEF1 promoter | This study |
| pJR165 | pRS415-DEF1-LexA-FLAG3-DEF1 3’UTR | DEF1-LexA | This study |
| pSH18-34 | LexAop(8)-GAL1p-LACZ URA3 2μ | Lex operator reporter | Golemis, 2001 |
|  | LexA-TBP-HIS3 | LexA-TBP expression vector | Chatterjee, S. |

**Supplemental Table 2: plasmids**

**References:**

Chatterjee, S., Struhl, K. Connecting a promoter-bound protein to TBP bypasses the need for a transcriptional activation domain. *Nature* **374**, 820–822 (1995).

Golemis EA, Serebriiskii I, Finley RL Jr, Kolonin MG, Gyuris J, Brent R. Interaction trap/two-hybrid system to identify interacting proteins. Curr Protoc Cell Biol. 2001 May;Chapter 17:Unit 17.3. doi: 10.1002/0471143030.cb1703s08.

Sweet T, Kovalak C, Coller J. The DEAD-box protein Dhh1 promotes decapping by slowing ribosome movement. PLoS Biol. 2012;10(6):e1001342. doi: 10.1371/journal.pbio.1001342. Epub 2012 Jun 12.

Pfannenstein J, Tyryshkin M, Gulden ME, Doud EH, Mosley AL, Reese JC. Characterization of BioID tagging systems in budding yeast and exploring the interactome of the Ccr4-Not complex. bioRxiv 2024 May 10:2024.05.09.593354. doi: 10.1101/2024.05.09.593354.
