# Supplementary material for: Gene expression analysis and proximity labeling reveal post-transcriptional functions of the yeast RNA polymerase II regulator Def1
Source: J Biol Chem. 2025 Dec 5;302(2):111003. doi: 10.1016/j.jbc.2025.111003 (PMC12804109; doi:10.1016/j.jbc.2025.111003)
Supplement: Supplemental Figures and Legends [file mmc3.pdf]

Supplementary Figure 1

A.

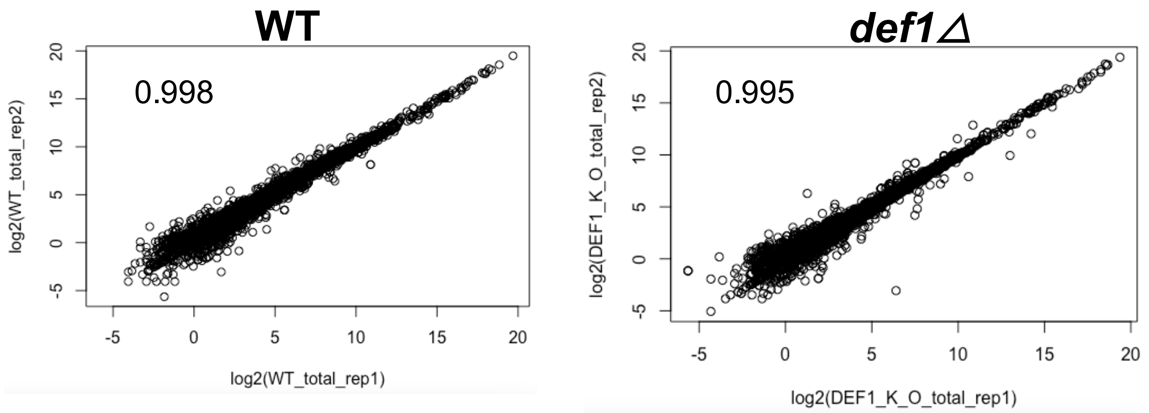

B.

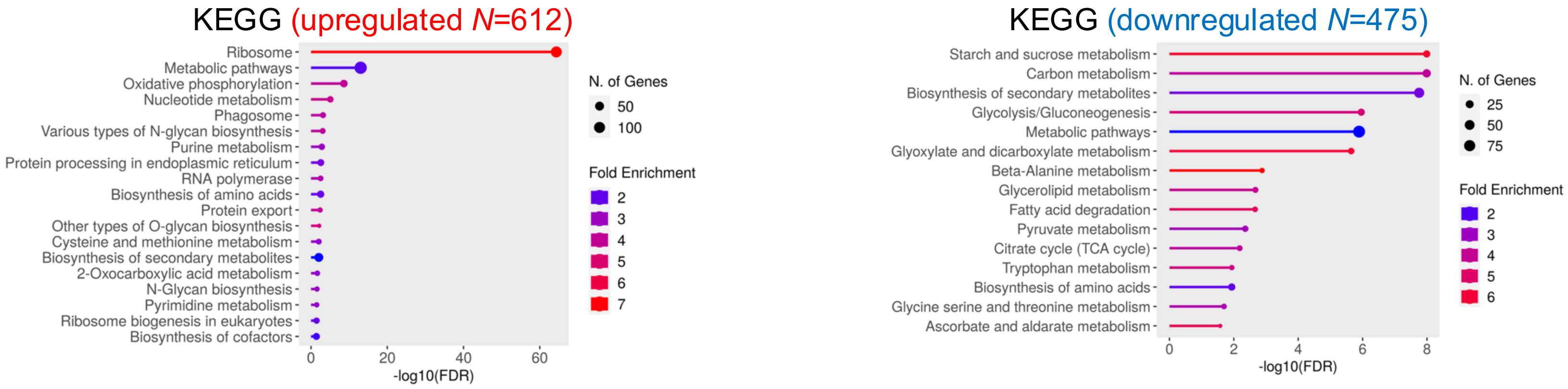

Supplementary Figure 2

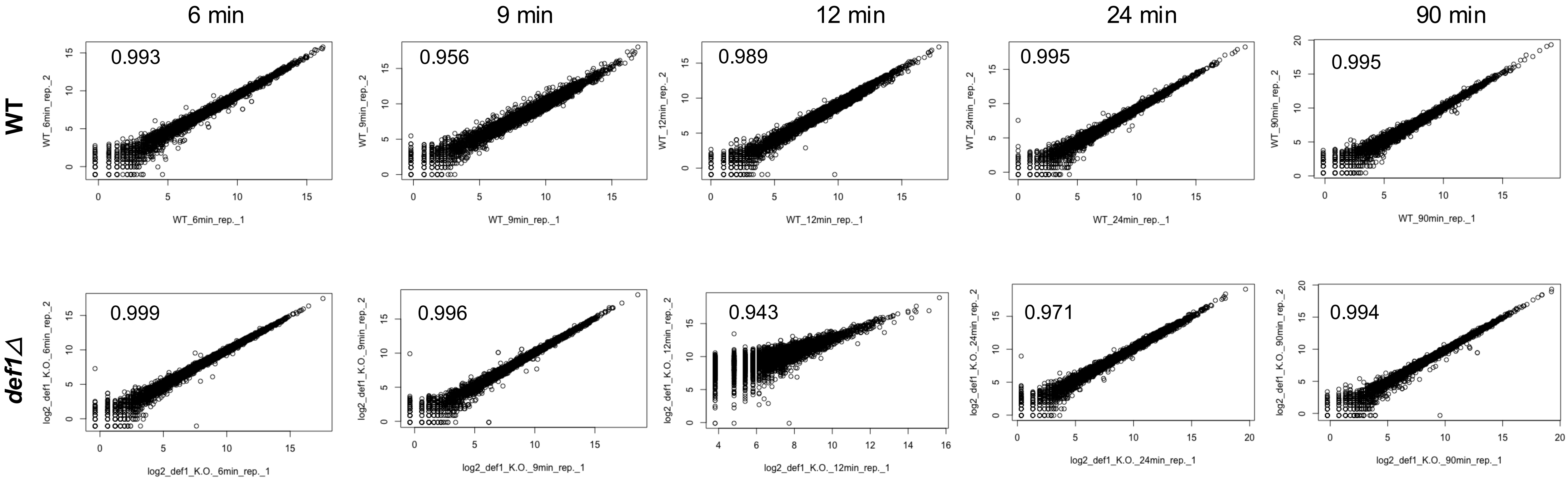

Supplementary Figure 3

**Article**	**Approach**	**Median halflife**
Miller *et al*.	DTA (4sU, 5mM)	11 min
Munchel *et al*.	4TU pulse-chase (4TU, 0.2mM)	18 min
Neymotin *et al*.	RATE-seq (4TU, 0.5mM)	10 min
Presynak *et al*.	RNAPII inactivation	7.4 min
Chan *et al*.	4-TU pulse only (4TU, 1mM)	3.6 min
Alalam *et al*.	SLAM-seq (4TU, 0.2mM)	9.4 min
Baudrimont et al.	Promoter shutdown	2 min
This study	RATE-seq (4TU, 5 mM)	2.7 min

Supplementary Figure 4

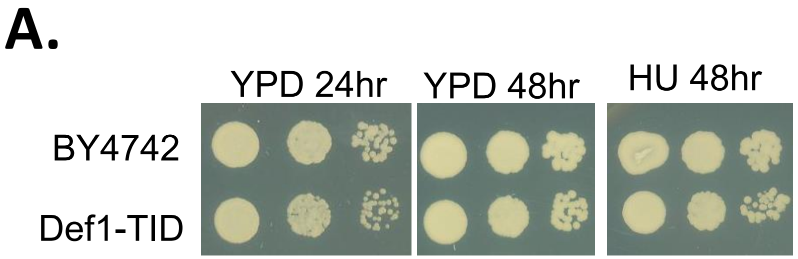

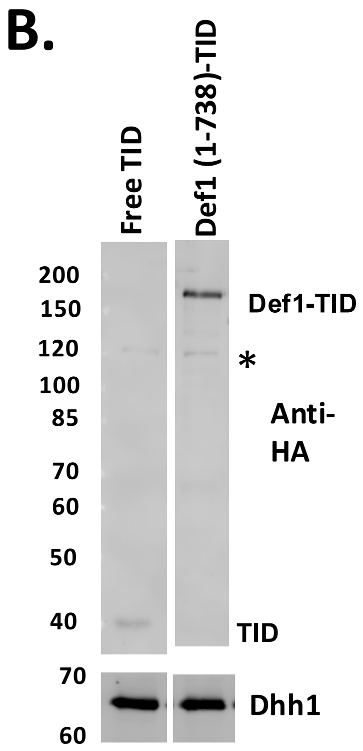

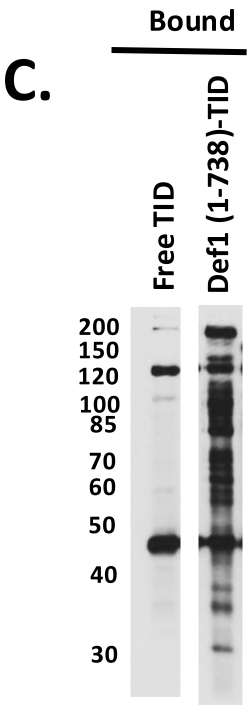

Supplementary figure 5

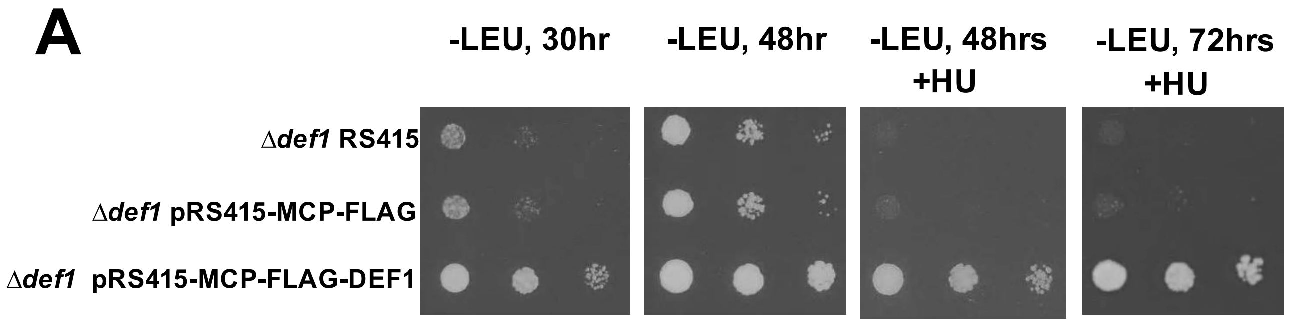

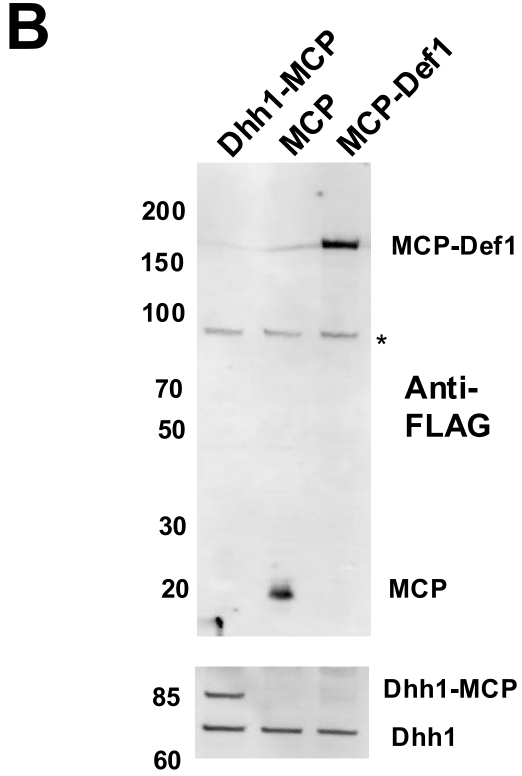

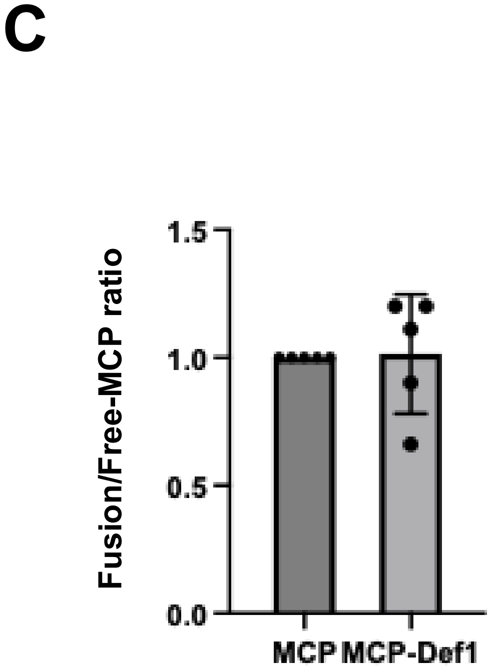

Supplementary figure 6

A

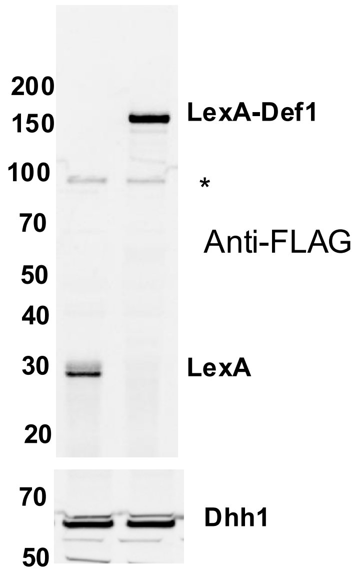

B

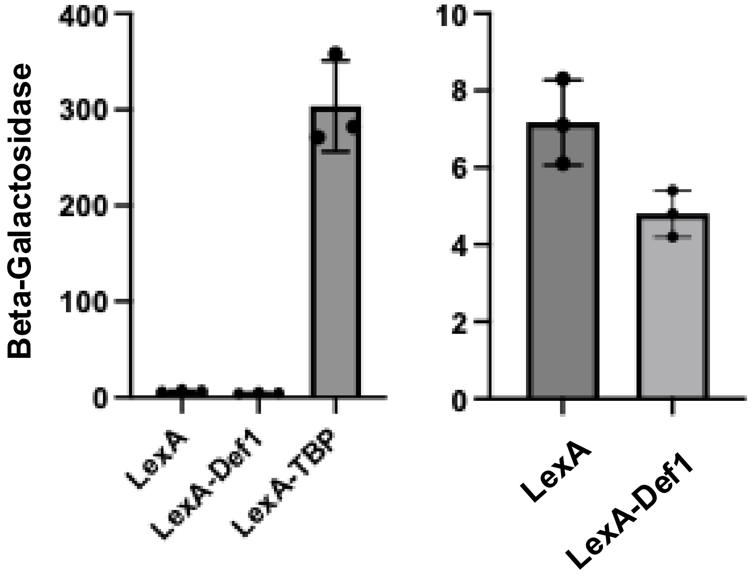

Supplemental figure legends

Supplemental Figure 1. (A). Correlation of biological replicates of total RNA-seq reads. The reads (FPKM) from biological replicates of each strain were plotted in a scatterplot. Pearson correlation coefficients were determined for the reads. ShinyGO 0.8 was used to identify GO terms for KEGG pathways of transcripts with an FC>2, and $p_{adj}<0.01$

Supplemental Figure 2. Correlation of RATE-seq reads between biological replicates. RATE-seq reads from biological replicates at each labeling time point (6 min, 9 min, 12 min, 24 min, 90 min) were plotted on a scatterplot. Pearson correlation coefficients between replicates were calculated and displayed on the respective plots.

Supplemental Figure 3. Comparison of the median RNA half-life from this and other studies. The differences in the methods and conditions are shown for comparison, since this can affect the value estimated from the studies. (MILLER et al. 2011; MUNCHEL et al. 2011; NEYMOTIN et al. 2014; PRESNYAK et al. 2015; BAUDRIMONT et al. 2017; CHAN et al. 2018; ALALAM et al. 2022).

Supplemental figure 4. Turbo-ID shows that Def1 labels proteins in the cell. (A). Spot test of wild type (BY4742) and Def1-TID-3HA cells (JR2200). Serial dilutions of saturated cultures were spotted onto YAPD and YAPD+ 75 mM hydroxyurea (HU). (B). Western blot of free TID-3HA expressed from the *CHA1* promoter (JR1951) and Def1-TID-3HA (JR2200). Dhh1 is used as a loading control. The asterisk indicates a protein cross-reacting with the HA antibody. (C). A representative streptavidin-pull-down. Proteins were eluted with SDS-PAGE loading buffer containing 3 mM biotin, separated on SDS-PAGE gels, and transferred to nitrocellulose. Biotinylated proteins were then detected using streptavidin-HRP.

Supplemental Figure 5: Supporting information for mRNA and promoter tethering experiments. (A). Complementation assay. A *def1*Δ strain (JR2204) was transformed with the plasmids indicated on the left. Cells were serially diluted and spotted on the

media indicated above the panel. HU was used at a concentration of 75 mM. (B). Representative western blot of cells expressing MCP, MCP-Def1 and Dhh1-MCP. The upper blot was probed with anti-FLAG (M2). The lower blot was detected using anti-Dhh1 antibody. Asterisk marks a cross-reacting band. A 3X-FLAG epitope was incorporated into the MCP and MCP-Def1 fusion proteins. Dhh1-MCP is not FLAG tagged and is not detected by the M2 antibody. (C). Quantification of the expression of the MCP fusion proteins. The proteins were detected using anti-FLAG antibodies. Total amounts of protein were controlled for by blotting for Taf14. The average expression of the MCP-Def1 fusion protein, relative to the amount of free MCP, which was set to 1.0. Average with standard deviations (N=5) is presented.

Supplemental Figure 6. Promoter tethering assay. (A). Anti-FLAG western blot. Dhh1 is the loading control (B). LexA- transcription activation reporter assay. (left) A Beta-galactosidase reporter gene driven by the *GAL1* core promoter containing 8 lexA binding sites (GOLEMIS et al. 2001) was co-transformed with plasmids expressing "free" LexA , LexA-Def1, and LexA-TATA-binding protein (TBP). LexA-TBP is a positive control, which drives expression by increasing PIC formation (CHATTERJEE AND STRUHL 1995) Three biological replicates were analyzed. (right) Plotting only the free LexA and LexA-Def1 derivatives to display the details on Beta-galactosidase expression.

References:

Alalam, H., J. A. Zepeda-Martinez and P. Sunnerhagen, 2022 Global SLAM-seq for accurate mRNA decay determination and identification of NMD targets. RNA 28**:** 905-915.

Baudrimont, A., S. Voegeli, E. C. Viloria, F. Stritt, M. Lenon *et al.*, 2017 Multiplexed gene control reveals rapid mRNA turnover. Sci Adv 3**:** e1700006.

Chan, L. Y., C. F. Mugler, S. Heinrich, P. Vallotton and K. Weis, 2018 Non-invasive measurement of mRNA decay reveals translation initiation as the major determinant of mRNA stability. Elife 7.

Chatterjee, S., and K. Struhl, 1995 Connecting a promoter-bound protein to TBP bypasses the need for a transcriptional activation domain. Nature 374**:** 820-822.

Golemis, E. A., I. Serebriiskii, R. L. Finley, Jr., M. G. Kolonin, J. Gyuris *et al.*, 2001 Interaction trap/two-hybrid system to identify interacting proteins. Curr Protoc Cell Biol Chapter 17**:** Unit 17 13.

Jiang, H., M. Wolgast, L. M. Beebe and J. C. Reese, 2019 Ccr4-Not maintains genomic integrity by controlling the ubiquitylation and degradation of arrested RNAPII. Genes Dev 33**:** 705-717.

Li,F., Xing,X., Xiao,Z., Xu,G. and Yang,X. (2020) RiboMiner: a toolset for mining multi-dimensional features of the translatome with ribosome profiling data. *BMC Bioinformatics*, **21**, 340.

Miller, C., B. Schwalb, K. Maier, D. Schulz, S. Dumcke *et al.*, 2011 Dynamic transcriptome analysis measures rates of mRNA synthesis and decay in yeast. Mol Syst Biol 7**:** 458.

Munchel, S. E., R. K. Shultzaberger, N. Takizawa and K. Weis, 2011 Dynamic profiling of mRNA turnover reveals gene-specific and system-wide regulation of mRNA decay. Mol Biol Cell 22**:** 2787-2795.

Neymotin, B., R. Athanasiadou and D. Gresham, 2014 Determination of in vivo RNA kinetics using RATE-seq. RNA 20**:** 1645-1652.

Pfannenstein J, T. M., Gulden ME, Doud, EH, Mosley, AL and Reese, JC, 2024 Characterization of BioID tagging systems in budding yeast and exploring the interactome of the Ccr4-Not complex. [preprint] bioRxiv.

Presnyak, V., N. Alhusaini, Y. H. Chen, S. Martin, N. Morris *et al.*, 2015 Codon optimality is a major determinant of mRNA stability. Cell 160**:** 1111-1124.

Wilson, M. D., M. Harreman, M. Taschner, J. Reid, J. Walker *et al.*, 2013 Proteasome-Mediated Processing of Def1, a Critical Step in the Cellular Response to Transcription Stress. Cell 154**:** 983-995.
